# Mining collections of compounds with Screening Assistant 2

**DOI:** 10.1186/1758-2946-4-20

**Published:** 2012-08-31

**Authors:** Vincent Le Guilloux, Alban Arrault, Lionel Colliandre, Stéphane Bourg, Philippe Vayer, Luc Morin-Allory

**Affiliations:** 1 Institut de Chimie Organique et Analytique (ICOA), Université d’Orléans, UMR CNRS 7311 B.P. 6759, rue de Chartres, 45067 Orléans Cedex 2, France; 2 Bioinformatic Modelling Department, Technologie Servier, 45007 Orléans Cedex 1, France; 3 Fédération de Recherche, Physique et Chimie du Vivant, Université d’Orléans-CNRS; FR 2708, avenue Charles Sadron, 45071 Orléans Cedex 2, France

**Keywords:** Chemical libraries, Molecular diversity, DRCS

## Abstract

**Background:**

High-throughput screening assays have become the starting point of many drug discovery programs for large pharmaceutical companies as well as academic organisations. Despite the increasing throughput of screening technologies, the almost infinite chemical space remains out of reach, calling for tools dedicated to the analysis and selection of the compound collections intended to be screened.

**Results:**

We present Screening Assistant 2 (SA2), an open-source JAVA software dedicated to the storage and analysis of small to very large chemical libraries. SA2 stores unique molecules in a MySQL database, and encapsulates several chemoinformatics methods, among which: providers management, interactive visualisation, scaffold analysis, diverse subset creation, descriptors calculation, sub-structure / SMART search, similarity search and filtering. We illustrate the use of SA2 by analysing the composition of a database of 15 million compounds collected from 73 providers, in terms of scaffolds, frameworks, and undesired properties as defined by recently proposed HTS SMARTS filters. We also show how the software can be used to create diverse libraries based on existing ones.

**Conclusions:**

Screening Assistant 2 is a user-friendly, open-source software that can be used to manage collections of compounds and perform simple to advanced chemoinformatics analyses. Its modular design and growing documentation facilitate the addition of new functionalities, calling for contributions from the community. The software can be downloaded at http://sa2.sourceforge.net/.

## Background

Exploring biology through the activity of small molecules is an established paradigm used in drug research for several decades now [1,2]. Today, a state of the art drug discovery program often begins with screening campaigns aiming at the identification of novel biologically active molecules. In the recent years, the rise of High Throughput Screening (HTS), combinatorial chemistry and the availability of large compound collections has led to a dramatic increase in the size of screening libraries, for both private companies and public organisations [3,4]. Yet, despite these constantly increasing capabilities, various authors have stressed the need to design better instead of larger screening libraries [5-9]. Chemical space is indeed known to be almost infinite, and selecting the appropriate regions to explore for the problem at hand remains a challenging task.

What we call library design and analysis actually aims to increase the likelihood of screening collections to contain potentially active compounds, while ensuring that any of these represent an acceptable starting point for lead optimisation. In terms of biological activity, the concept of molecular diversity has proven useful to design libraries containing diverse chemotypes and hence increase the ratio of hits [10,11]. In terms of resource optimisation, the main difficulty lies in the removal of those nuisance compounds that are unlikely to be developed into effective drugs, especially using biochemical assays. For instance, reactives, warheads, promiscuous aggregating inhibitors, or more simply non-drug-like compounds are usually filtered out to avoid a waste of resources [12,13]. Most of these undesired properties are usually represented by either simple rule-based flags derived from physico-chemical properties (e.g. the Lipinski Rule of 5 [14]), or structural features encoded using the SMARTS notation [15].

The domain of chemoinformatics provides a plethora of methods that can be used to tackle various aspects of library analysis. Despite the growing diversity of available modeling and chemoinformatics tools, there are few software tools specifically dedicated to the management and the analysis of screening libraries. One of the main reasons for this no doubt is the specificity of each screening platform (e.g. plates format, automatic collection of results), which requires more specific developments to collect the results and associate them with tested molecules. Typically, screening collections are stored internally using in-house, usually web-based proprietary software programmes, some of which have been described in the literature [16-18]. Other general-purpose, proprietary packages also propose methods to handle chemical libraries. In particular, the Instant JChem application [19] from Chemaxon encapsulates various chemoinformatics functionalities, and makes it possible to store libraries in a database environment. Another example in this category is the CACTVS toolkit [20], an extensible distributed client/server system for the computation, management, analysis and visualisation of chemical information.

In the open-source literature, there is a growing range of tools that can deal with chemical libraries, each of them having its own specific applicability domain, ranging from general purpose to highly specific software. Workflow solutions allow the automation of numerous recurrent tasks, such as data reading / writing, filtering or visualisation, and some of them integrate chemistry functionalities. KNIME [21] in particular, is distributed with a set of chemistry nodes available for the Chemistry Development Kit (CDK) [22,23] and other chemoinformatics packages such as RDKit [24] and Indigo [25]. Various other advanced features have been encapsulated in KNIME nodes, and it is thus possible to e.g. compute molecular descriptors, perform substructure searches, or extract scaffolds. Recently, the CDK was also integrated in the Taverna workflow solution [26,27] through a set of more than 160 different workers handling chemoinformatics tasks. The combination of chemoinformatics functionalities with data-mining methods typically available in workflow solutions makes it possible to use more advanced strategies to analyse the content of chemical libraries.

Other more general-purpose software tools have been recently published by the chemoinformatics open-source community. Bioclipse [28] for example, is a general purpose modeling software that combines bioinformatics and chemoinformatics functionalities in a modular and extensible workbench. In the context of the present work, Bioclipse can be used to perform simple routine tasks such as chemical file reading and visualisation, descriptor calculation, and SMART matching. More closely related to this work, AMBIT XT [29,30] is a chemoinformatics data management software, which consists in a MySQL database and a set of functional modules, allowing a variety of queries, data mining and predictive model building and application. Although not specifically dedicated to the management of screening libraries, it contains a set of chemoinformatics and data-mining facilities that make it usable for analysing collections of compounds. The software was recently enhanced by providing a set of OpenTox API [31] compliant REST web service interfaces to most of its functionalities, hence promoting collaborative development and data sharing [32]. More specific tools are clearly beyong the scope of this work, but it is worth mentioning the Scaffold Hunter [33], which allows one to display a chemical library in the form of an interactive scaffold tree, or the SARANEA package [34], which allows one to derive similarity graphs that can be used to perform structure-activity Relationship analysis.

This article presents Screening Assistant 2 (SA2), an open-source desktop software for chemical library management. SA2 stores unique chemical structures and properties in a MySQL database, and allows a variety of advanced chemoinformatics analyses and datamining queries to be performed. It has been designed to handle small to very large (up to millions of molecules) collections, and to integrate external sparse data in a flexible way (e.g. molecular descriptors, biological activities..). Besides various search and visualisation capabilities, SA2 can also be used to manage the provenance of stored compounds, and to create new subsets of molecules using many different methods, e.g. filtering, merging or diversity. SA2 was developed for facilitating the analysis of screening libraries, and to regroup multiple ways of mining these collections using chemoinformatics methods. Broadly speaking, it can also be used to quickly and interactively analyse the content of any chemical dataset.

## Implementation

SA2 is open-source software, which means complete transparency and scalability in terms of algorithm implementation. A first version of the software was available on sourceforge and on the web-site of our laboratory [35,36]. This new version has been re-designed from scratch, keeping most of the concepts and features that were available in the first version. In this section, the general architecture of the software will be described, as well as the most important features and algorithms that make up its originality.

### Architecture

SA2 is a desktop application based on the NetBeans Platform [37], a generic framework for JAVA / SWING based software. The NetBeans Platform allows applications to be developed in a modular fashion, thereby promoting good software engineering and programming practices. It contains a set of basic modules that can be used to handle various aspects of software development (e.g. fully dockable windowing system, module versioning or automatic updates), that would be otherwise time-consuming to (re)develop. This modular achitecture makes it easier to add new menus, actions, extension points and modules without the need to go deep into the existing source code of the application. It is written in pure JAVA, and is therefore expected to be crossplatform. So far, it has been succesfully tested on Windows XP and 7, Linux Ubuntu 10.4, CentOS 5. Issues related to the NetBeans Platform were found on some MacOSX operating systems which make the software currently incompatible with it.

### Storage engine

All the data are stored in a MySQL database [38] using the InnoDB engine, which ensures data integrity. MySQL is a widely adopted database engine, extensively documented, and with a wide user community. The choice of a database engine makes it possible to manage very large libraries - a database of around 7 million unique molecules has been successfully set up in our lab - and perform various routine tasks (e.g. filtering) more efficiently that using a simple file system. A mandatory requirement when using SA2 is therefore to have a MySQL server [38] installed and running on a server (or a simple desktop computer) that can be reached through a local network, along with a valid user account.

### Input / Output

**Figure 1:**
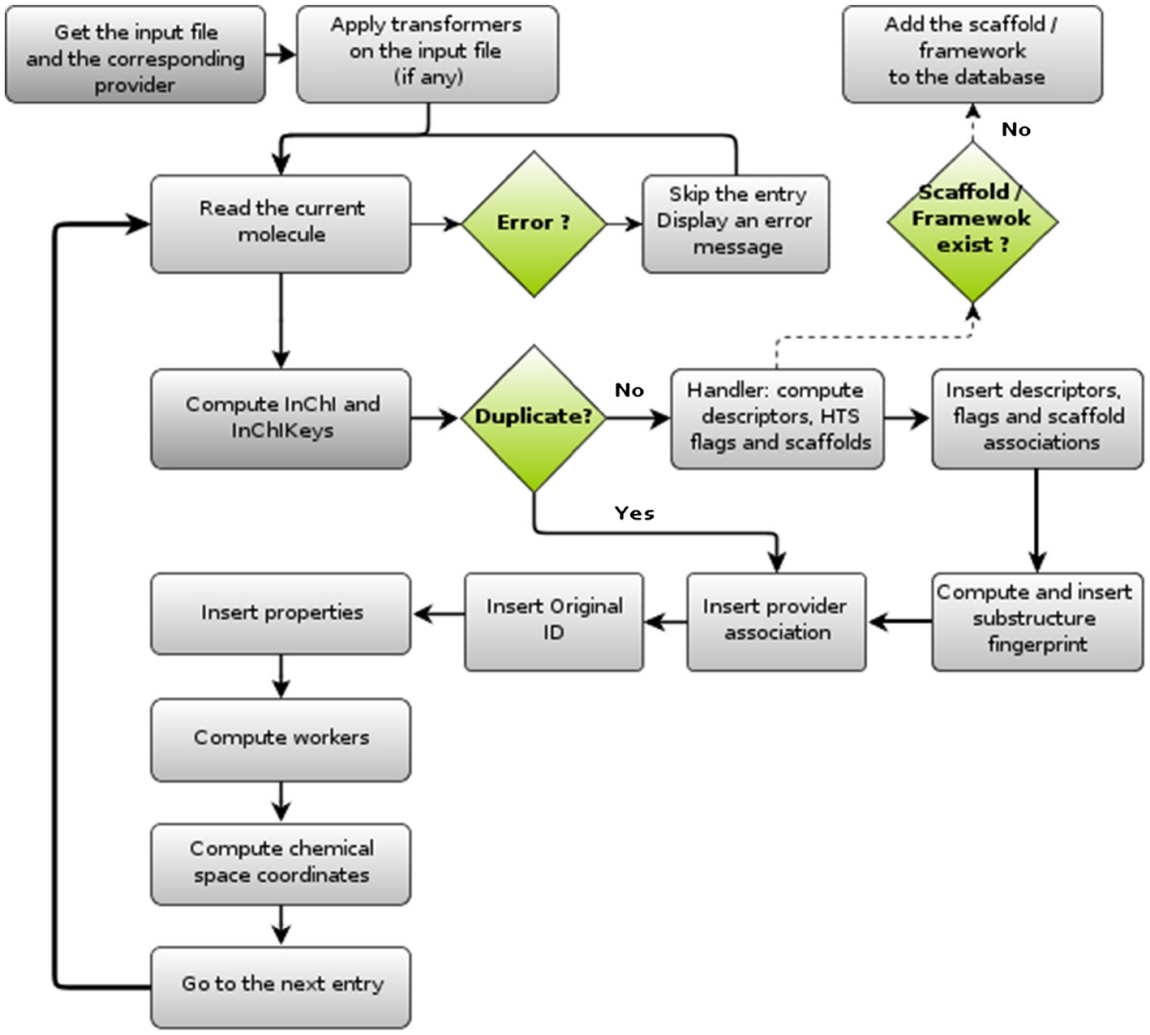
Full workflow of the SDF import process in SA2.

SA2 databases must be fed with MDL Mol-formatted input files (.sd or .sdf files) containing the full structure of the molecules. The full import workflow is shown in Figure 1. A step-by-step wizard is available to help new molecules import (and properties), as described in the documentation. Original molecules’ names, if any, can be associated with each molecule, along with their CAS number. An internal unique database ID will also be automatically generated for each molecule. Text-delimited input files are also supported, and any number / kind of property can be associated with each existing molecule. These properties are stored in existing or new tables / fields that can be created either directly during the import process, or using the dedicated window in SA2. The entire database (or a subset of it - see *Providers and libraries*) can be exported in the MDL Mol-format and / or in text-delimited format.

### Storing and perceiving molecules

SA2 stores unique molecules under the form of a connection table in MDL Mol-format. Duplicates are removed using the IUPAC InChI identifier v1.0.4, taking into account stereochemistry. In the standard version of SA2, no particular pre-processing is applied on the input molecules, i.e. salts are not removed, protonation states and stereochemistry are kept as defined in the input file, etc. Although the standardisation of molecules is of prime importance, this procedure is usually problem-dependent, and we chose not to introduce any particular (possibly undesired) modification on the input molecules. It is however possible to code one’s own standardisation services, refered to as ”Transformers”, that will be executed in a defined order - see the developer documentation for further details.

When a new set of molecules is imported into a database, various properties are automatically computed and associated with each molecule. To this end, SA2 introduces the notion of Molecular handler. A Molecular handler is an entity (typically a chemistry-aware programming library) that is responsible for the perception of molecules. For each newly imported molecule, the handler reads the molecule and perceives atom types, calculates some simple properties (listed in Table 1), computes the scaffold and framework of the molecule, and computes various HTS-related flags. A molecule that cannot be loaded by the handler (e.g. invalid format) will not be imported into the database (an error message informs the user on each problematic molecule). Each database must be associated with a particular handler, which cannot be changed once the database has been created. Two different handlers are available in the current version of SA2: JOELib handler and the CDK handler, respectively based on the JOELib library [39] and the CDK library [22,23]. For computational performance reasons, the JOELib handler is used by default for new databases.

**Table 1:** List of properties and flags that are automatically calculated when importing new molecules

**Type**	**Name**	**Description**	**Ref.**
SMARTS	Reactive	Reactive compounds (15)	[12]
	Warhead	Warhead compounds (20)	[12]
	PAINS <15	Pan Assay Interference Compounds (409)	[13]
	PAINS <150	Pan Assay Interference Compounds (55)	[13]
	PAINS >150	Pan Assay Interference Compounds (16)	[13]
Flags	RO5	Lipinski’s rule of 5 *	[14]
	RO3	Fragment rule of 3 *	[40]
	Exotic	Unrecognised atom type *	-
	Salt	Disconnected structures	-
Descriptors	Weight	Molecular weight	-
	LogP	Caculated logP *	-
	Heavy atoms	Number of heavy atoms	-
	HBA	Number of hydrogen bond acceptors *	-
	HBD	Number of hydrogen bond donors *	-
	Halogens	Number of halogen atoms	-
	Rot. Bonds	Number of rotatable bonds *	-
	Ring count	Number of rings (SSSR)	-
	Max Ring size	Maximum size of rings	-

The definition of descriptors marked by an asterisk is handler-specific.

Table 1 provides the full list of properties that are computed for new molecules. Simple physicochemical descriptors will systematically be computed and stored in the main table of the database. Several binary flags can also be computed to provide simple ways of filtering compounds using widely adopted rules. The Lipinski drug-like [14] flag and the Rule of 3 flag [40] will be associated with each molecule. A set of 5 SMARTS-based flags is also available to provide warnings on potentially problematic compounds that typically contain undesired substructure patterns. Reactive and warhead compounds as defined in [12] are flagged as such. Three additional flags which account for Pan Assay Interference Compounds (PAINS) are also available. These filters were defined in a recent paper [13] that highlights the need to remove a variety of classes of compound that are likely to be characterised as false positives in biochemical screening. The SMARTS version of these classes of compounds was extracted by Rajarshi Guha and made available to the community on his blog [41].

SMARTS-based flags are defined by a set of SMARTS queries which are stored in the database. The value of each flag for each molecule will be 1 if the molecule matches any of the SMARTS for a particular flag, 0 otherwise. The user can add / deactivate / delete any of these SMARTS, and hence recalculate the value of all the flags at any moment. An additional flag was also created to contain only user-defined SMARTS queries.

### Properties and fingerprints

There are basically two ways of storing properties and fingerprints in SA2: importing them from an external source, and computing them directly within SA2, when possible. Properties and fingerprints in SA2 are organised in tables, and each table is assigned to a category. Various tables are available in the default version of SA2, e.g. tables for CDK descriptors / fingerprints and or MOE [42] descriptors. For new properties, the user must create new tables, either manually using the appropriate wizard, or directly when importing new molecules / properties. Fingerprints on the other hand, can only be imported using text-delimited files, in an fingerprint table which must be created using a dedicated editor prior to the import process. Two input fingerprint formats are currently supported: simple binary strings, and index strings where only the index of each bit set to 1 is recorded.

Several descriptors can also be computed directly within SA2. These include almost all the descriptors and fingerprints available in the CDK, several descriptors based on the JOELib library that were already available in the previous version of Screening Assistant, the Klekota and Roth fingerprint derived from [43], and the Indigo similarity fingerprint [25]. All these descriptors / fingerprints are stored in pre-installed tables. The reader should also note that two tables are available for MOE descriptors, which must however be computed externally and imported back into the database using SDF or text files. These two tables are automatically inserted because various PCA-based chemical spaces available in SA2 are based on these descriptors (see [44] and the next sections for details).

### Providers and Libraries

When importing a new set of molecules, the user will be asked to associate these molecules with a so-called *provider*. A *provider* was primarily intended to represent commercial vendors that propose collections of compounds. In practice however, there is no restriction on what a *provider* can represent. The notion of *provider* can hence be thought of as ”where your compounds come from”, e.g. a commercial vendor, a specific medicinal chemistry project, etc.

*libraries* on the other hand, represent subsets of molecules within a particular SA2 database. There are many ways of creating / modifying *libraries*: simple filtering rules using any descriptor(s), merging two existing libraries, creating a diverse library, creating a scaffold-based library, or saving search results. This concept is probably the most important one in SA2, as it provides great flexibility in the management of new sets of molecules. Once a new library has been created, it can be further analysed using the various visualisation and chemoinformatics facilities included in SA2, or simply exported in any output format available. Moreover, many tasks (e.g. searches, creation of diverse subsets) can be performed either on the whole database, or restricted to a particular library.

### Scaffolds / Frameworks

A scaffold is a particular substructure that can be obtained based on the full structure of a query molecule. The best known Scaffold definition is that of Bemis and Murcko [45], who defined the scaffold of a molecule as being the union of ring systems and linkers.

In SA2, each newly imported molecule is associated with a unique Scaffold and a unique Framework. The scaffold retains all rings and linkers between rings, and removes all lateral chains, with the exception of exocyclic double bonds. The Framework of a molecule is the same as the scaffold, except that atom types are removed and bond orders are set to 1, leaving all atoms to SP3 carbons. The only exception here is 6-membered aromatic rings, for which the bond order is kept to retain aromaticity [35,36]. All remaining lateral chains, resuling from the exocyclic double bonds kept in the scaffold, are also removed.

Two windows in SA2 are dedicated to displaying the scaffold / framework of a molecule selected in any other window. All the molecules that belong to a particular scaffold can then be easily saved as a new library, added to an existing library, or removed from the database. Scaffolds are also used by diversity selection algorithms available in SA2, as described in the following sections. A report can also be generated, which will retrieve the most populated scaffolds, along with other information such as the total number of scaffolds in the database, the average number of molecules per scaffold and so on. This report can be generated for the whole database or for a particular library.

### Visualisation

The concept of chemical space has been widely adopted by the chemoinformatics community as a way to represent and compare sets of molecules. SA2 provides various ways of visualising multiple molecules. Simple X-Y plots can be used to draw molecules in an interactive panel using two selected properties. A similarity graph view can also be used, whereby molecules are drawn in the form of a graph. Nodes then represent molecules, and edges are drawn between two nodes if the corresponding molecules have a similarity higher than a given threshold.

SA2 also encapsulates the Delimited Reference Chemical Subspaces (DRCS) methodology described in a recent article [44]. DRCS are defined by the combination of a Principal Component Analysis (PCA) model (DRCS model), and one or several subspace(s) delimitation(s) (DRCS contour) intended to encompass the most populated part spanned by a particular library. This delimitation is computed on a reduced (2D) space obtained using the PCA model, and is based on the calculation of an average convex hull. Isolated compounds (outliers) are excluded prior to the creation of this delimitation, which finally represents the most populated (dense) subspace spanned by the reference library.

Three pre-computed DRCS models are available in new SA2 databases. These DRCS have been described in [44], and make use of different sets of descriptors. For each model, several subspaces are also included, which represent the densest part of different types of collections, e.g. general purpose HTS molecules, Oprea Leadlike molecules [46], Pharmaceutical molecules or fragment molecules (Rule of 3 [40]).

### Molecular diversity

SA2 provides the possibility of extracting diverse subsets of molecules using scaffold-based min-max algorithms. The diversity selection can be performed on the whole database or on an existing library. This way, one can restrict the search to a carefully selected set of molecules. Briefly, the base algorithm is designed to ensure the presence of one molecule per scaffold (or framework, depending on the user’s choice). It starts by retrieving all scaffolds within the database (or the selected library). These scaffolds are either randomly shuffled, or ordered by decreasing number of associated molecules. The first molecule is added to the library as being the molecule that is the most similar to an average fingerprint computed on all the molecules that belong to the first selected scaffold. The similarity between two molecules is defined by any similarity coefficient (e.g. Tanimoto) available in SA2 applied to the selected fingerprint. Next, for each remaining scaffold, the molecule having the lowest similarity to the currently selected molecules is added to the library.

A maximum similarity cutoff can also be defined. For a particular scaffold, all candidate molecules that have a similarity to the already selected molecules greater than this cutoff are not accepted, thereby ensuring that similar scaffolds are not over-represented in the library. The counter part of this is a higher computational complexity if the similarity cutoff is defined too small.

Once all the scaffolds have been processed, the final number of molecules may still be lower than the desired size of the library. Two reasons can lead to this situation: (1) the number of scaffolds in the database is lower than the required number of molecules, and (2) the similarity cutoff used is too small. In both cases, the entire selection process is just repeated. In the second case, the cutoff is automatically increased for each new run. The selection process finally stops when *N* molecules have been selected.

### Searches

Various search capabilities are available in the software, such as exact structure, similarity, substructure, SMARTS searches, or simpler searches using the name or database ID. Similarity searches can be performed using any of the fingerprints available in the database. The entire database is scanned within the application, and a bitwise comparison is performed using the selected similarity metric (Tanimoto coefficient by default). A similarity search using a query molecule that does not exist in the database nevertheless requires the use of a fingerprint that can be calculated within SA2 (i.e. not an external fingerprint that has been imported into the database). SMARTS search is performed by retrieving molecules from the database and applying a SMARTS matching algorithm (referred to as SMARTS engine in the application) to detect matches. It is also possible, with a working internet connection, to visualise a SMARTS query using the SMARTS viewer service provided by the bioinformatics center of the university of Hamburg [47,48].

Substructure search is performed as a two-step process, with a prescreening step followed by a graph isomorphism test. The first step is done using a database query that filters out the molecules that cannot match the query. This query makes use of two different types of information: (1) a small set of basic properties (number of heavy atoms, number of SSSR, and number of halogens) that are indexed in the main table, and (2) a fingerprint that is calculated for each molecule upon import. This fingerprint is computed using the Indigo library, which provides an implementation of a specific substructure fingerprint. The fingerprint is encoded as a set of 33 unsigned integers of 32 bits, which means that a maximum of 1056 bits is accepted. The fingerprint calculation can be replaced by one’s own implementation (see Extension points), and the values associated with each molecule can be subsequently updated by recomputing the fingerprint on the entire database.

For any type of search, the results obtained are displayed in a specific window, and can be saved as a new library, added to an existing library, or removed from the database.

### Extension points

The Netbeans Platform makes it possible to define extension points, referred to as Services, using a NetBeans Platform-specific mechanism. A Service is a JAVA class that is able to provide a specific functionality as defined by the Service facade it corresponds to (a JAVA interface). For example, a *FingerprintSimilarityMetric* service provides a floating number ranging from 0 to 1, based on two fingerprints representing two molecules. The *SubstructureFingerprint* service on the other hand, returns a fingerprint based on a SDF text representing a molecule. Based on such a mechanism, one can easily add new services by simply implementing the corresponding service interface, and registering it using a single line annotation. Various extensions points were actually mentioned previously, e.g. molecular handlers, transformers, SMARTS engine, substructure fingerprint, or substructure engine. The only requirement to setup your own service implementation is to know the full list of services along with their specifications, and provide an implementation of it in a new module; there is usually no need to change or even know the source-code of the original application that makes use of this service. For example, in a new SA2 module, adding a new similarity metric can be performed by adding a single JAVA class implementing the corresponding interface, i.e:

@ServiceProvider(service=FingerprintMetric.**class**)

**public class **FPTanimotoMetric **implements**

FingerprintMetric

 { 

@Override

**public **String getName() {

**return **"Tanimoto" ;

 }

@Override

**public **String getDescription() {

**return **"Tanimoto___coefficient___for___

fingerprints." ;

 }

**public float **getSim(BitSet m1, BitSet m2) { 

// *Implementation*...

 }

 (..)

 }

The most important line here is the @ServiceProvider. Using this JAVA annotation, one register this class as a fingerprint metric service. No more operation is required except implementing the methods defined by the FingerprintMetric interface: the new metric will automatically appear everywhere the FingerprintMetric service is required, e.g. for similarity search, for diverse subset creation.. The developer documentation of SA2 provides detailed examples on this.

One particular service should be emphasised here. The *MolWorker* service can be implemented to compute any operation of an input molecule. MolWorkers can be activated either directly when importing new molecules, or can be run afterwards using the dedicated menu. This service offers the possibility of adding any additional calculation in a completely transparent way. From the point of view of a developer, the only requirement (as for all services) is to create a new module, and to provide a registered implementation of the MolWorker interface. There is no restriction on the operation that a MolWorker should do. In the 1.0 version of SA2, three workers are available: the CDK worker, which calculates CDK fingerprints and descriptors, the JOELib worker, which does the same using the JOELib library, and the Indigo worker, which makes it possible to compute two fingerprints.

## Results

To illustrate the use of SA2, various collections of different sizes have been analysed. A brief description of the Graphical User Interface (GUI) is first provided as an introduction. In the first case study, an in-house database of 6.3 million unique, standardised (see [44] for details) compounds collected from 73 commercial vendors was used. In the second case study, two focused commercial screening libraries were compared, and SA2 was used to select a diverse subset from a combination of the two libraries. Finally, various possible improvements and perspectives will be discussed. All the data and figures presented here have been generated with the version 1.1.0 RC1 of SA2.

### Graphical User Interface (GUI)

**Figure 2:**
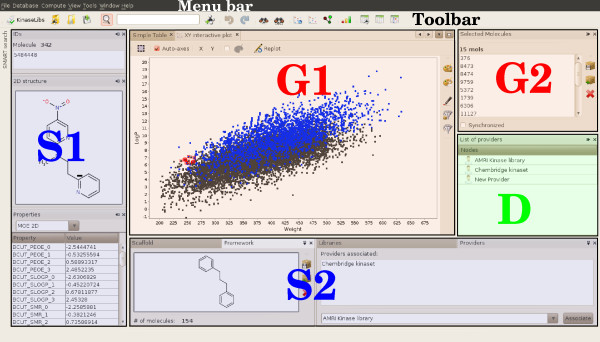
**Graphical User Interface overview.** Three types of windows are highlighted here: S1 and S2 in blue show a set of Singleton windows that display the information on single molecules. G1 and G2 (red) are two group windows. G1 is an interactive X-Y plot where the Chembridge (dark gray) and AMRI kinase libraries (blue) are plotted. G2 represents the list of selected molecules that are highlighted in G1. D in (green) is a database window that displays the list of providers.

An overview of the GUI is given in Figure 2. The GUI is composed of four main types of window: (1) Singleton windows, where the information on a single molecule is displayed (e.g. 2D structure, list of associated providers..); (2) Group windows, where multiple molecules can be displayed in a certain form (e.g. 2D plot, simple tables..); (3) Database windows, which usually display a list of specific entities stored in the database (e.g. providers, libraries, SMART flags..); and (4) other windows which do not fit into any of these categories. All the windows are accessible through the main menu located at the top of the main window, and shortcuts are also available for various views in the toolbar. Each window can be opened, closed, reduced, undocked or maximised, which provides great flexibility in selecting the relevant information that needs to be displayed. The relative positioning of windows is also completely customisable using intuitive drag and drop, and is restored upon startup.

In Group windows, the user can select the molecules of its choice interactively. When a single molecule is selected, each Singleton window is updated to display the information corresponding to this molecule. When multiple molecules are selected, they are highlighted in the corresponding view, and a specific window is updated to display the list of molecules that forms the current selection (as illustrated in Figure 2). Based on this list, various operations can be performed, such as removing these molecules from the database, or creating a new library based on this selection. It is also possible to synchronise the selection, whereby molecules selected in one Group window are automatically selected in all the other Group windows that are open.

Most of the algorithms are available in the Compute menu. This menu includes all the advanced algorithms and search capabilities that can be applied on the database, such as diversity selection, creation of new DRCS models, similarity search, etc.

### Case study 1: large scale analysis

A collection of 73 SDF files representing 73 different commercial vendors was imported into an SA2 database. The precise collection and pre-processing of these data is described elsewhere [44]. It took around 4 weeks to import all the data into the database. This was mainly due to the large number of molecules to be processed (15 million altogether): for each molecule, more than 400 SMARTS have to be matched, dozens of descriptors are calculated, the scaffolds and frameworks are computed along with their InChI and InChIKey, and all these data must then be stored and indexed within the MySQL database.

**Figure 3:**
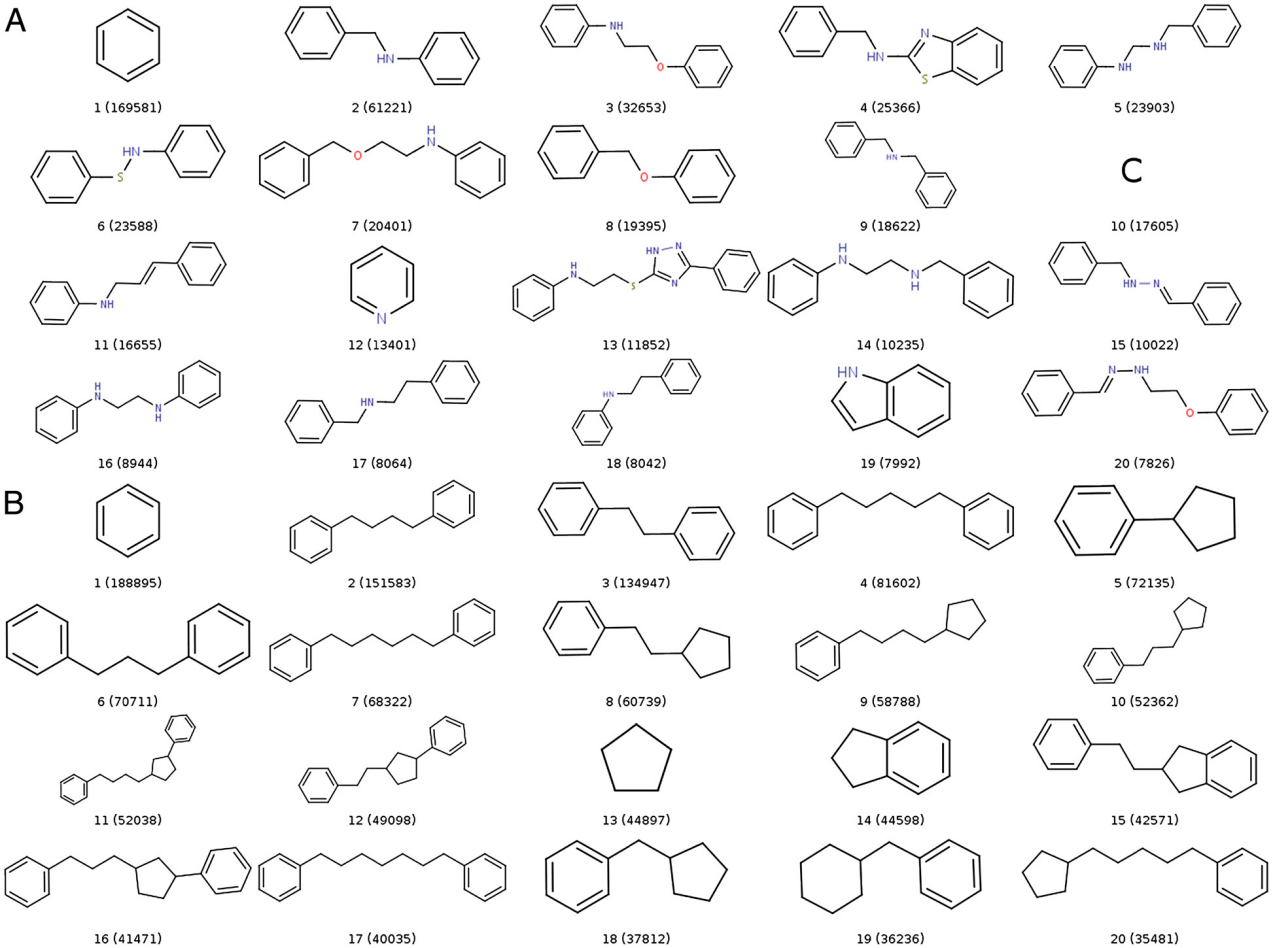
**List of the 20 most populated scaffolds (A) and frameworks (B) in the database of 6.7M compounds.** The number of compounds associated with each core structure are displayed in brackets. These pictures were generated using the Scaffold report of SA2.

In this illustrative analysis, the basic properties and HTS flags listed in Table 1 were computed, as well as the scaffolds and frameworks of each molecule. Various reports were generated within SA2, and the results are summarised in Tables 2, 3, 4, Additional file 1: Table S1 and S2 for detailed values, and in Figure 3.

**Table 2:** Scaffold and framework representativity

	**Scaffold**		**Framework**	
	**Count**	**Percent**	**Count**	**Percent**
Database	1 084 411	15.24%	247 689	3.47%
Singleton	647 260	9%	104 250	1.5%
Cumulative freq. (50%)	11 565	1.06%	447	0.18%
Cumulative freq. (80%)	150 777	13.87%	7 741	3.13%

Singletons are core structures that are associated with only one molecule. Percentages for Database and Singleton rows are expressed as the number of scaffolds (Count column) divided by the total number of molecules in the database. Cumulative frequency values represent the number (resp. proportion) of scaffolds that are needed to obtain a certain percentage of compounds (in brackets) in the database.

**Table 3:** Summary of the scaffold composition and unicity analysis

		**Scaffolds**		**Frameworks**	
	**Unicity (CU)**	**Proportion**	**Unicity (SU)**	**Proportion**	**Unicity (FU)**
Min	0%	6.3%	0%	2.2%	0%
Max	100%	84.2%	87.9%	57.8%	49%
Average	24.9%	24.7%	13.3%	11.6%	5.9%
> 10%	37	69	27	26	13
> 20%	33	37	17	11	8
> 50%	15	4	4	1	0

Unicity is defined as the proportion of molecules (or scaffolds / frameworks) that are exclusive to a given provider, i.e. that cannot be found in any other provider. The proportion of scaffolds / frameworks are expressed as the number of molecules divided by the number of scaffolds / frameworks associated with a given provider. The minimum, maximum and average values through all vendors are given in this table. The number of vendors having one of these indices up to a given threshold is given in the second part of the table.

**Table 4:** Summary of the drug-like analysis

				**Undesired**	
	**Fragment-like**	**Non-Drug-like**	**Reactive**	**PAINS**	**Global**
Min	1.3%	0%	0%	0%	0%
Max	86.7%	25.6%	19.5%	27.5%	29%
Average	27.7%	5.7%	6.9%	5.9%	12.3%
> 5%	59	36	36	35	66
> 10%	47	13	21	14	48
> 20%	34	1	0	1	7

Percentages are expressed as the minimum, maximum and average proportion of molecules that are flagged for each criterion, computed over each provider. The number of providers having one of these indices up to a given threshold is given in the second part of the table.

#### Scaffolds and frameworks

Table 2 outlines the results obtained by generating a Scaffold and a Framework report in SA2. A total of 1 084 411 scaffolds and 247 689 frameworks can be found in the database, representing respectively 15.24% and 3.47% of the compounds. Only 1.06% and 0.18% of the scaffolds are needed to obtain 50% of the compounds in the database, which shows that only a very few scaffolds and frameworks represent a very large part of the database. Figure 3 shows the 20 most populated scaffolds and frameworks as extracted from the Scaffold report of SA2. Interestingly, the Benzene core structure is the most populated for both scaffolds and frameworks, with almost 3% of the compounds associated with it. There is also a significant proportion of acyclic compounds, which are represented here by a single carbon atom. On the other hand, the uneven distribution of scaffolds and frameworks is highlighted with 59.52% and 42.09% of scaffolds / frameworks which are associated with a single molecule. This corresponds to 9% and 1.5% of the molecules in the database that have a unique scaffold / framework.

Additional file 1: Table S2 presents the detailed results in terms of compound unicity, and scaffolds / frameworks composition for each provider. An outline of these results can be found in Table 3. The reader should note that low percentages of compounds unicity (CU), scaffold unicity (SU) and Framework unicity (FU) have to be analysed carefully: some providers include the libraries of other providers in their own collection, which obviously biases the results. Large unicity values on the other hand are more informative.

As summarised in Table 3, around half of the providers have a CU greater than 10%, and only 15 greater than 50%. In terms of the proportion of scaffolds and frameworks, almost all providers contain at least 10% of unique scaffolds, but this number drops to only 26 providers having more than 10% of frameworks, which suggests that some providers have a significant proportion of scaffolds that share the same graph and differ only in their heteroatoms composition. On average, providers contain 24.7% of scaffolds and 11.6% of frameworks. Providers with the largest proportion of scaffolds are generally those that contain a small number of compounds. There are however, some top-populated providers that have a large proportion of scaffolds and high unicity as well, e.g. ChemDiv (CU = 54.97%, SU = 45.23% and FU = 31.49%). The ChimiothèqueNationale, which federates collections of synthesis products available in French academic laboratories, also contains a large percentage of original compounds, (CU = 85.24%, SU = 64.23% and FU = 37.37%), hence highlighting the potential interest of academic screening collections.

#### Drug-likeness

The database was also analysed in terms of Drug-like properties. Various reports were generated with SA2 for the Lipinski rule of 5, the fragment Rule of 3, Reactive and PAINS flags. The detailed results can be found in Additional file 1: Table S2, and an outline is provided in Table 4. These data show that there is a fairly low percentage of potentially problematic compounds in screening libraries. On average, 5.7% of compounds fail the Rule of 5, while 6.9% of compounds are found reactive and 5.9% might be PAINS compounds. These average values nevertheless mask some differences between providers, with some of them having up to 20% percent of potentially problematic compounds. The percentage of fragment-like compounds is on the other hand more evenly distributed, with many providers containing more than 50% of fragments. Besides the availability of libraries specifically designed for fragment-based screening, these high percentages can also be explained by the presence of building blocks. Providers containing a large number of molecules usually contain a small percentage of fragment-like compounds (the 15 providers containing the largest number of molecules have less than 10% of fragments), which are much more heavily represented in small to medium-sized libraries.

#### Conclusion

In conclusion, a detailed picture of the composition of compound collections from various points of view can be obtained using SA2. In this example, a large number of available collections was analysed in terms of scaffolds and drug-like properties. Such an analysis can be used to provide a detailed picture of the chemical space spanned by the available screening libraries, and to identify those collections that can be of great interest in terms of originality.

### Case study 2: kinase libraries

For this second illustrative example, we assume the need to create a diverse subset of potential kinase inhibitors based on two existing focussed libraries: the AMRI kinase library (3232 molecules), and the Chembridge Kinase library (11501 molecules). Both libraries were downloaded from their respective vendors’ websites, pre-processed as described in [44], and imported into a SA2 database (a different provider were associated with each input file), leading to 14732 unique molecules. SA2 was subsequently used to analyse both libraries in order to guide the creation of a diverse subset that contains 1500 molecules (around 10% of the entire database).

**Figure 4:**
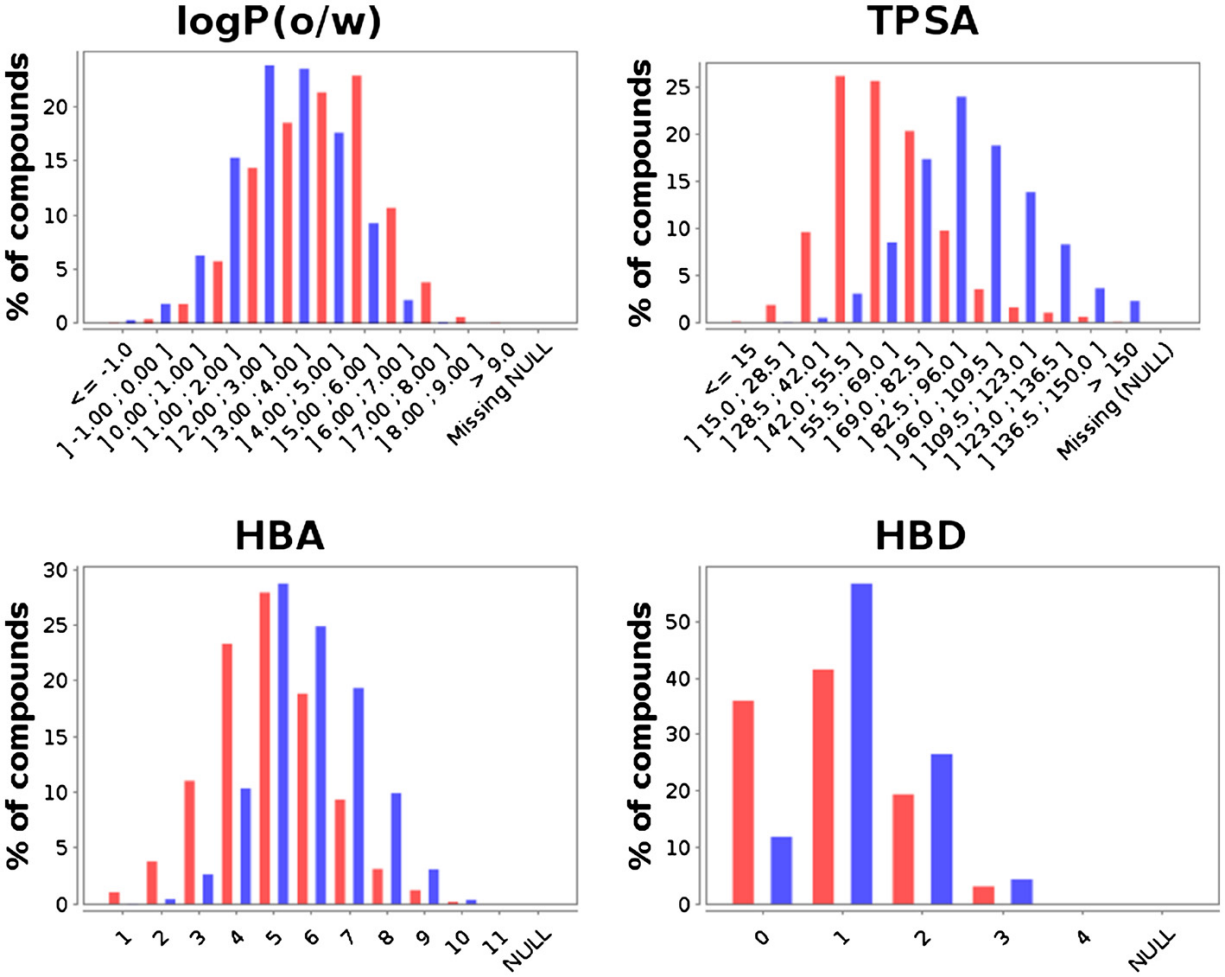
**Comparative distribution of some physico-chemical properties.** Chembridge Kinaset (red) and the AMRI kinase library (blue). HBA (resp. HBD) stands for Hydrogen Bond Acceptor (resp. Donor). These histograms can be obtained by simply clicking on the property to analyse in the *Properties* window of SA2.

**Figure 5:**
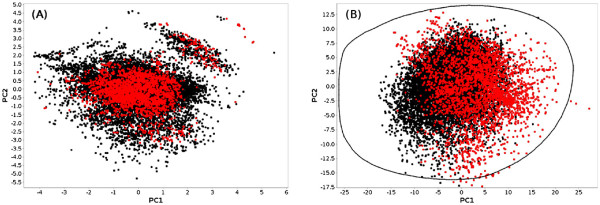
**PCA projections of the Chembridge (black dots) and AMRI (red dots) libraries.** The first reduced space (**A**) has been computed within SA2 on the entire kinase database using the CDK BCUT descriptors which were computed upon import. The second reduced space (**B**) is the DRCS-MOE2D space which is already available in new SA2 databases, and for which descriptor values were imported. The contour shown in black encompasses the densest region spanned by HTS compounds (see [44] for details).

A simple analysis was first conducted to compare the distribution of various physico-chemical descriptors in the two libraries. Using the Property Stats window of SA2, the distribution of any property can be plotted and compared between any set of libraries (or on the entire database). Figure 4 shows a comparison of the distribution of some physico-chemical descriptors for both libraries. What can be concluded from this simple analysis is that the two libraries seem fairly complementary, with some properties showing different distributions. The same observation can be made when comparing the scaffold composition of the two libraries. The Charts window was used here to evaluate the overlap between the libraries in terms of compounds and scaffolds. Table 5 shows that there is a small overlap between the two libraries in terms of compounds, scaffolds and frameworks. Only one compound is shared between the two libraries, and they both contain a large percentage of exclusive scaffolds and frameworks. All these observations are further supported by the projection of both libraries in reduced chemical spaces. Figure 5 shows the projection of the two libraries in a PCA space computed on the entire database using the CDK BCUT descriptors, and in the DRCS-MOE2D reduced space. Despite some visible overlaps, there are obviously some parts of each space that are covered by only one of the two SA libraries.

**Table 5:** Scaffold, framework and compound originality of the AMRI and Chembridge kinase libraries

	**AMRI**		**Chembridge**	
	**Count**	**Percent**	**Count**	**Percent**
Frameworks	873	27.0%	2 204	19.2%
Frameworks unicity	747	85.6%	2 078	94.3%
Scaffolds	1 053	32.6%	4 036	35.1%
Scaffolds unicity	1 008	95.7%	3 991	98.9%
Overlap (compounds)	1 molecule			

The proportion of scaffolds (resp. frameworks) is expressed as the number of unique scaffolds (resp. frameworks) divided by the number of molecules in the library. The scaffolds (resp. frameworks) unicity is expressed as the number of scaffolds (resp. frameworks) unique to the library divided by the total number of scaffolds (resp. frameworks) in the library.

The first step in creating a diverse library is to remove all the potentially problematic compounds. To this end, two filtered libraries were created for each provider (i.e. each original library). The filters were defined to remove all reactive, warhead and PAINS compounds in both libraries. The AMRI and Chembridge libraries contained respectively 159 and 587 such compounds. A third library was then created by merging the two previously created libraries. This new library was subsequently used to create a scaffold-based diverse subset of 1500 molecules using the default parameters (scaffold-based, MACCS fingerprint, Tanimoto metric with a similarity cutoff of 0.6 increased by 0.1 at each new run, frequency-based ordering of core structure). The diverse subset creation procedure took around 4 seconds to complete.

**Figure 6:**
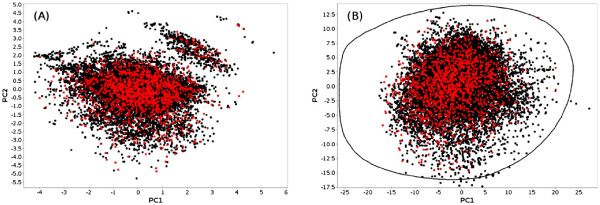
**PCA projections of the diverse subset (red dots) in the two spaces described previously.** The remaining molecules (AMRI + Chembridge) are drawn in black. The figure has been generated with the *DRCS plot* window of SA2.

Once a new diverse subset has been obtained, SA2 offers various ways of assessing its diversity. A first overview can be obtained by plotting the entire database and highlighting the subset in one or several reduced spaces available in SA2. Figure 6 shows the projection of the diverse library within the two spaces used previously. Such a plot provides a good overview of the chemical space coverage of our library. One can easily see that it covers the space spanned by the entire database fairly well, although some parts of the space remain rather poorly represented (Figure 6b). This can be explained by the fact that there is a significant difference in the size of the two original libraries. As a consequence, the Chembridge diverset was clearly oversampled. The composition of the diverse subsets which contain 78% of molecules coming from the Chembridge library. A simple way to obtain a more balanced selection would be to extract a random subset from the Chembridge library, and perform the diversity selection on the union of this random subset and the AMRI library. Repeating the process to test this hypothesis, a new library was obtained which contained 60% of Chembridge compounds. As suggested by the remaining bias, it seems that overall, the Chembridge library offers more diversity.

Several reports available in SA2 can provide further insight into the diversity of a library. In particular, the Similarity report (*Compute–Similarity–Similarity report*) can be used to calculate various diversity indices based on any of the fingerprints available in SA2, along with their distribution. The percentage of scaffolds can also be easily obtained, as shown previously. Table 6 shows various diversity indices computed in SA2 on the diverse library, and two random subsets of the same size. Obviously, the diverse library shows greater diversity in terms of scaffolds, frameworks, and similarity. The difference between random and diverse subsets becomes however less visible when using different fingerprints than that used to create the diverse subset. This behavior is to a certain extent expected as the diverse subset has been optimised using the MACCS fingerprint. The diverse library however, remains the most diverse in all cases, and in particular for the average neirest neighbor (Avg. NN) similarity indice, which is known to be efficient in discriminating between to libraries of the same size [49].

**Table 6:** Diversity evaluation for diverse and random subsets

	**Diverse**	**Random 1**	**Random 2**
Scaffold%	84%	61%	63%
Framework%	52%	44%	44%
MACCS			
Avg. pairwise	0.44	0.48	0.48
Avg. NN	0.76	0.88	0.88
Max. sim.	0.80	1.00	1.00
Pubchem			
Avg. pairwise	0.48	0.50	0.50
Avg. NN	0.82	0.87	0.87
Max. sim.	0.98	1.00	1.00
Indigo			
Avg. pairwise	0.26	0.29	0.29
Avg. NN	0.70	0.81	0.81
Max. sim.	1.00	1.00	1.00

The percentage of scaffolds and frameworks are reported for each library. The Tanimoto metric and three different fingerprints were also used to compute average pairwise similarity (Avg. pairwise), average nearest neighbor similarity (Avg. NN), and maximum pairwise similarity (Max. sim) within each library, using 3 different fingerprints that can be computed directly within SA2. These data were generated with the *Similarity report* and the *Scaffold report* of SA2.

### Discussion

#### Performance and possible improvements

An SA2 database is indexed and optimized to obtain a good compromise between the time needed to import new molecules, and the time needed to perform all the analyses available in the software. Most of the calculations are therefore quite fast: plotting several tens of thousands of molecules in a reduced or an X-Y plot can be achieved within few seconds depending on the hardware, keeping the interactive selection completely functional. The scaffold and framework reports illustrated in the “Results” section can be obtained within around two minutes for 7 million compounds. For newly imported molecules, and despite the large number of calculations involved, the process is fast enough to rapidly set up new databases for small to medium-sized datasets. Of course, the time needed to import new molecules will increase with the number of workers selected. From this point of view, the only possible improvement would be to split each input file and distribute the computation through several threads. Although it does not pose any substantial technical challenge, special care would nevertheless be required as to the integrity of the data as the database will be accessed by multiple processes. The diverse subset algorithms also performs very efficiently. As illustrated previously, a diverse subset can be obtained rapidly using the default parameters. This performance is due to the heuristic nature of the algorithm, which was designed to obtain a good compromise between an optimum diversity and an algorithm that can be used on very large databases. The performance of the algorithm obviously depends on the number of molecules required as well as the similarity cutoff used. Higher value of the similarity cutoff lead to more diverse libraries, but require more time to complete.

Despite the use of a database engine as a backend storage solution, SA2 is not a chemistry database cartridge, nor it is based on any particular existing one. Despite this matter of fact, most of the search capabilities of SA2 also perform fairly well. The name and exact structure search are fully optimised, and the results can be retrieved almost immediately, even for very large databases. Similarity search is of course slower, but the performance is still acceptable. A similarity search launched in medium-sized databases (e.g. 100 000 molecules) retrieves the results within one or two seconds, depending on the hardware. Similarity searches in the 7 million database, using the Tanimoto metric (cutoff = 0.7) and the Indigo similarity fingerprint (512 bits), took less than 3 minutes to scan the entire database on a modern computer through a local network. Typically, around 50 000 structures per seconds are processed for such a search. Furthermore, the search results are updated for each new hit, making it possible to browse them as they are retrieved. The performance of similarity search actually depends on three main factors, in decreasing order of importance: the similarity cutoff (the lower it is, the slower the search will be, as much more results have to be retrieved), the size of the fingerprint used, and the location of the database.

Substructure search has also been optimised, but is still subject to some limitations in specific cases. For instance, the pre-filtering step can be almost useless if the query substructure is too generic, e.g. a simple benzene. Using scaffolds provides a way to retrieve a subset of the results almost immediately, but the remaining of the database still have to be scanned. Two possible improvements could be rapidly made to accelerate substructure search: (1) improve the fingerprint used as filtering, but this would not solve the problem mentioned previously and (2) store a set of generic small structures, and flag all compounds containing these structures when importing them into the database. This second solution may provide the advantage of having the results of low-specificity substructure searches directly available. On the other hand, it would certainly impact the time needed to import new molecules, and require a substantial amount of additional disk space. Finally, although it would require more development efforts, the integration of an existing chemoinformatics database cartridge, such as Bingo [50], MyChem [51], PgChem [52] or OrChem [53], may certainly represent an interesting direction. As SA2 currently only support MySQL, it would be of particular interest to integrate cartridges for other database engine, e.g. Bingo or OrChem for PostgreSQL.

#### Perspectives

SA2 was initially designed to manage and analyse screening libraries. The most important missing piece is probably the possibility to manage screening projects (including plates, activity types, targets..), and to integrate the results of HTS assays in a more specific way. The diversity of HTS assays and screening results types pose substantial challenges as to the organisation and integration of the resulting data. Currently, SA2 offers the possibility of associating any kind of property to each molecule, but in a non-specific and uncontrolled way. This provides great flexibility, but it would no doubt be advantageous to integrate controled vocabulary (typically an ontology) in order to organise the data more appropriately. In a recent article, Visser et al. [54] described a novel approach to standardise, organise and semantically define biological assays and screening results. Ontologies can be truly valuable in the mining of HTS data, and open up exciting perspectives for tools like Screening Assistant 2.

Although at first restricted to the domain of screening, SA2 is also moving toward becoming a more general-purpose software that deals with chemical libraries in a broad sense. Hence, there is again considerable room for adding new chemoinformatics, datamining or Structure-activity relationship features that are more specific to the analysis of small to medium-sized datasets. The first step toward this was taken by adding a graph similarity view (see “Implementation” section, or the official documentation), which is primarily useful to perform SAR on small (one or two thousand molecules) datasets. A Self-organizing map module is also on its way, which will provide a complementary non-linear method to the PCA currently available in SA2.

## Conclusions

Screening Assistant 2 complements the growing ecosystem of modeling tools by providing a set of chemoinformatics facilities integrated in a database environment. It facilitates the management of chemical libraries through an intuitive and interactive graphical interface, and provides a set of advanced methods to analyse and exploit their content. As with any new software, there are still many improvements that can be made, and probably even more directions to take. Special care was taken to provide a comprehensive documentation for both users and developers. We therefore encourage anyone to feed the project with remarks, new ideas and features, and hope that the software will be useful to the community.

## Availability and requirements

**Project name:** SA2

**Project home page:**http://sa2.sourceforge.net/

**Operating system(s):** Platform independent

**Programming language:** JAVA / SQL

**Other requirements:** Java 1.6.0 or higher http://java.sun.com/, MySQL 5.1 or higher http://dev.mysql.com/downloads/mysql/, and the NetBeans Platform 6.9.1 for developers willing to add new modules http://netbeans.org/features/platform/.

## License

Screening Assistant 2 is released under the terms of the GNU General Public License as published by the Free Software Foundation; either version 2 of the License, or (at your option) any later version.

## Competing interests

The authors declare that they have no competing interests.

## Authors’ contributions

VLG designed and implemented the software, and drafted most of the manuscript. LMA initiated and supervised the development of Screening Assistant. PV and AA supervised specific parts of the project, fed it with new ideas, and participated in testing the software. LC and SB made extensive tests, and participated in documenting the software. All authors approved the final manuscript.

## Supplementary Material

Additional file 1 **sumpinf/providers.pdf.** Two large tables containing detailed values for the provider analysis. Click here for file
