# Supplementary material for: Mining collections of compounds with Screening Assistant 2
Source: J Cheminform. 2012 Aug 31;4:20. doi: 10.1186/1758-2946-4-20 (PMC3547782; doi:10.1186/1758-2946-4-20)
Supplement: Additional file 1 — sumpinf/providers.pdf. Two large tables containing detailed values for the provider analysis. [file 1758-2946-4-20-S1.pdf]

Mining collections of compounds with Screening Assistant 2

Vincent Le Guilloux*[1], Alban Arrault[2], Lionel Colliandre[1], Stéphane Bourg[3], Philippe Vayer[2], and Luc Morin-Allory*[1]

[1] Institut de Chimie Organique et Analytique (ICOA), Université d'Orléans, UMR CNRS 7311 B.P. 6759, rue de Chartres, 45067 Orléans Cedex 2, France.
[2] Bioinformatic Modelling Department, Technologie Servier, 45007 Orléans Cedex 1, France.
[3] Fédération de Recherche, Physique et Chimie du Vivant, Université d'Orléans-CNRS; FR 2708, avenue Charles Sadron, 45071 Orléans Cedex 2, France

Email: Vincent Le Guilloux*- vchem@users.sourceforge.net; Luc Morin-allory*- luc.morin-allory@univ-orleans.fr;

*Corresponding author

SUPPLEMENTARY INFORMATION

Table S1 - Proportion of flagged compounds by provider

Table S2 - Uniqueness and scaffold composition by provider

Table S1 - Proportion of flagged compounds by provider

The proportions are given for each provider, as the number of molecules that are flagged divided by the total number of molecules. The Undesired column represents the proportion of compounds that have been flagged by at least one of the 4 HTS flags (reactive and PAINS).

Provider	Size	RO5	RO3	Reactive	PAINS< 15	PAINS< 150	PAINS> 150	Undesired
Ambinter	5204292	92.75	5	2.81	0.23	1.19	4.46	8.38
Enamine	1516762	97.09	4.49	2.95	0.1	0.9	2.53	6.3
ScientificExchange	1184178	85.77	5.42	2.46	0.14	3.29	8.28	13.52
Princeton	971576	92.33	7.44	2.13	0.34	2.1	7.07	11.14
VitasMLab	839835	91.48	6.48	2.22	0.51	2.35	7.92	12.43
ChemDiv	663386	90.59	2.28	1.86	0.21	1.74	6.59	9.57
Chembridge	633235	97.72	7.26	1.7	0.61	1.35	5.6	8.89
Specs	471247	92.24	9.05	2.13	0.37	2.73	6.81	11.39
InterBioScreen	463396	92.86	5.11	2.56	0.24	2.39	8.95	13.22
Asinex	379455	92.44	6.21	1.72	0.25	1.59	4.78	8.02
LifeChemical	333633	96.22	3.44	1.26	0.06	0.41	3.61	5.19
AMRI	254611	74.4	1.4	1.41	0	0.64	3.93	5.9
AlindaChemical	253867	93.98	9.43	1.71	0.29	1.57	3.58	6.9
AsisChem	234085	91.4	4.44	1.72	0.36	4.05	10.86	16.07
Pharmeks	227343	86.91	3.86	3.1	0.34	2.41	10.79	15.72
TimTec	204835	92.66	13.62	3.47	0.65	2.19	5.97	11.76
ChemTI	170752	93.64	9.99	1.97	0.29	1.67	2.16	5.84
ChemicalBlock	125109	90.25	7.8	2.86	0.87	2.04	6.32	11.57
Otava	120101	95.72	6.89	2.27	0.08	1.78	6.67	10.39
Labotest	105237	94.14	20.73	5.37	0.34	3.04	8.45	16.34
Nanosyn	65041	93.52	12.51	2.41	0.43	2.99	9.25	14.28
Maybridge	60391	97.38	20.05	3.27	0.26	1.43	3.33	7.67
SynthonLab	52713	93.02	6.76	3.78	1.08	7.62	18.79	29.04
Key-Organics	42647	97.05	10.98	6.56	0.74	1.47	3.84	11.86
Bionet	42640	97.05	11.02	6.54	0.74	1.48	3.85	11.86
ChimiothequeNationale	41950	93	27.14	9.27	0.03	1.13	2.99	12.93
Apollo Scientific	38981	98.31	58.16	17.21	0.12	1.05	1.34	19.19
MatrixScientific	38321	99.43	55.11	12.52	0.11	2.37	2.59	17.1
FluoroChem	34377	98.37	59.1	13.94	0.1	1.22	1.18	16.17
Intermed	32041	81.26	1.33	0.09	0.03	2.82	7.37	9.57
Arkive	30511	95.16	35.31	6.33	0.79	0.87	5.76	13.03
EMC	28097	84.7	3.04	1.57	0	0.54	2.27	4.39
Analyticon Discovery	26427	85.9	2.26	3.76	0.02	1.52	2.38	7.5
IS-Chemical-Technology	25041	98.21	61.46	12.9	0.13	1.05	1.14	14.78
Aronis	23707	96.04	12.35	3.75	0.4	3.74	3.24	10.82
TOSLab	16557	80.92	2.87	2.55	1.55	4.28	11.45	18.11
Peakdale	14621	94.07	4.86	1.01	0.06	0.04	0.74	1.51
TorontoResearchChemicals	13249	88.5	25.87	12.41	0.1	1.44	2.88	16.27
Oakwood	13208	99.08	66.5	13.95	0.09	0.95	0.74	15.66

Table S1 (continued)

Provider	Size	RO5	RO3	Reactive	PAINS< 15	PAINS< 150	PAINS> 150	Undesired
ALBChemical	9998	99.19	10.37	2.91	0.03	1.82	9.48	13.64
ARVI	9960	98.65	33.99	8.43	0.13	1.6	2.4	11.9
MyriaScreen	9934	99.3	14.75	2.68	0.34	1.96	4.64	9.14
Spectrum	8673	90.89	4.44	2.43	0.07	2.4	8.68	13.48
CombiBlock	7087	99.84	59.22	4.74	0	0.08	0.17	4.98
Menai	4012	98.48	12.59	12.34	0.25	8.28	5.81	23.58
Sinova	3791	98.87	61.12	8.92	0.11	0.79	0.76	10.31
ChemiK	3760	99.92	83.32	19.34	0.08	0.72	0.27	20.11
ACBBlocks	3232	100	69.89	14.36	0	2.66	3.25	20.2
Szintekon	2664	98.46	39.08	10.47	0	2.06	2.55	14.08
PepTech	2376	99.03	24.33	1.73	0	0.17	0.63	2.53
FocusSynthesis	2321	98.54	55.75	8.32	0	0.34	0.52	9.09
ExclusiveChemistry	2272	89.74	24.34	3.65	0	0.53	1.63	5.5
Sequoia	2132	89.82	17.73	10.79	0.38	4.17	3.05	17.4
Biosynth	1804	91.19	37.31	12.58	0.11	1.83	1.66	15.63
Cayman	1777	88.91	14.24	18.8	0.06	2.59	1.8	22.85
FrontierScientific	1677	96.42	72.39	17.05	0	0.12	0	17.17
SynChem	1546	100	58.47	19.47	0.26	1.81	2.26	23.29
InFarmatik	1373	99.85	45.67	7.79	1.38	0.66	1.53	11.22
Biotrend	1261	87.63	19.11	5.55	0.16	4.04	3.81	12.61
Prestwick	1181	92.97	22.44	6.27	0.51	2.03	2.96	11.26
Adesis	1173	99.83	46.63	6.73	0	0.09	0	6.82
Chemivate	1108	98.74	1.71	1.71	0	0	0	1.71
Endeavour	813	99.88	81.55	14.88	0	0.49	1.11	15.62
Endotherm	677	91.43	33.68	11.23	0	1.92	0.15	13.15
KaironKem	647	100	58.27	14.22	0.31	5.26	5.72	23.18
GreenPharma	647	93.35	23.65	8.04	0	0.46	0.15	8.65
AFChemPharm	617	96.27	48.3	8.1	0.16	1.46	1.13	10.21
Chess	563	100	54.71	7.46	0	0.36	1.42	9.24
Synphabase	490	88.98	37.55	14.49	0.41	0.41	3.88	18.37
Pyxis	317	100	77.6	0	0	0	0.63	0.63
Sinof	263	100	86.69	13.69	0	0	0	13.69
EnzoLifeSciences	202	90.1	16.83	18.32	0	1.49	0	19.8
Azasynth	67	100	14.93	0	0	0	0	0

Table S2 - Uniqueness and scaffold composition by provider

Unicity is defined as the proportion of molecules (or scaffolds / frameworks) that are exclusive to a given provider (that cannot be found in any other provider). The proportion of scaffolds / frameworks are expressed as the number of scaffolds / frameworks divided by the number of molecules associated with a given provider.

			Scaffolds		Frameworks	
Provider	Size	Unicity (%)	Proportion (%)	Unicity (%)	Proportion (%)	Unicity (%)
Ambinter	5204292	29.41	16.63	14.23	3.88	11.09
Enamine	1516762	9.3	28.9	8.96	6.17	3.13
ScientificExchange	1184178	56.7	9.58	29.43	3.11	21.6
Princeton	971576	14.7	13.47	10.18	4.3	6.69
VitasMLab	839835	0.15	16.66	0.04	5.45	0.03
ChemDiv	663386	54.97	17.98	45.23	7.1	31.49
Chembridge	633235	2.79	25.05	3.9	7.9	1.04
Specs	471247	4.44	16.88	1.76	6.07	1.41
InterBioScreen	463396	1.41	18.67	1.41	6.93	0.3
Asinex	379455	36.13	20.8	34.13	7.47	22.26
LifeChemical	333633	2.77	18.25	3.78	6.48	1.02
AMRI	254611	1.09	11.5	0.31	6.23	0.1
AlindaChemical	253867	2.43	16.35	0.34	5.52	0.22
AsisChem	234085	14.31	12.37	4.69	4.82	1.92
Pharmeks	227343	0.02	23.11	0	9.93	0
TimTec	204835	0.65	25.94	0.36	9.38	0.17
ChemTI	170752	3.07	17.08	3.57	6.36	3.55
ChemicalBlock	125109	0.89	29.05	0.71	11.87	0.22
Otava	120101	10	17.83	4.77	7.3	1.64
Labotest	105237	36.94	24.44	20.99	8.86	9.33
Nanosyn	65041	20.07	23.6	10.14	9.61	5.33
Maybridge	60391	50.52	30.23	23.52	9.08	7.83
SynthonLab	52713	1.8	11.33	0.77	5.01	0.38
Key-Organics	42647	0.14	26.48	0.11	8.99	0.05
Bionet	42640	0	26.45	0	8.96	0
ChimiothequeNationale	41950	85.24	28.24	64.23	12.39	37.37
Apollo Scientific	38981	0.26	12.27	0	3.6	0
MatrixScientific	38321	13.39	10.76	4.63	2.48	1.89
FluoroChem	34377	20.69	10.36	8.54	3.2	6.73
Intermed	32041	0.02	7.05	0	3.7	0
Arkive	30511	0.09	28.85	0.05	11.42	0
EMC	28097	98.91	14.81	87.93	8	45.22
Analyticon Discovery	26427	0.01	32.09	0	15.79	0
IS-Chemical-Technology	25041	26.11	13.21	20.34	5.19	12.23
Aronis	23707	0	16.25	0	6.68	0
TOSLab	16557	0.49	36.2	0.2	21.18	0.11
Peakdale	14621	0	36.13	0	16.05	0
TorontoResearchChemicals	13249	64.87	27.07	39.18	14.15	27.25
Oakwood	13208	1.25	10.49	0.29	3.1	0.24

Table 2 (continued)

Provider	Size	Unicity (%)	Scaffolds		Frameworks	
			Proportion (%)	Unicity (%)	Proportion (%)	Unicity (%)
ALBChemical	9998	0.07	20.33	0	8.83	0
ARVI	9960	4.5	25.38	1.19	12.11	0.25
MyriaScreen	9934	11.15	45.14	5.73	18.7	0.75
Spectrum	8673	2.24	37.59	0.95	21.93	0.37
CombiBlock	7087	29.24	10.44	13.92	3.01	2.35
Menai	4012	0	33.85	0	16.13	0
Sinova	3791	62.49	11.37	14.62	6.07	4.35
ChemiK	3760	6.41	6.3	2.53	2.18	2.44
ACBBlocks	3232	1.39	18.25	1.02	6.03	0
Szintekon	2664	65.73	25.26	43.24	11.26	14.33
PepTech	2376	48.95	11.7	14.03	5.01	0
FocusSynthesis	2321	44.68	34.17	29.63	15.68	11.81
ExclusiveChemistry	2272	67.21	19.94	18.54	8.93	7.88
Sequoia	2132	22.84	44.93	6.58	27.77	4.05
Biosynth	1804	42.18	18.02	13.23	10.31	7.53
Cayman	1777	67.08	34.83	32.15	22.06	14.8
FrontierScientific	1677	20.57	14.13	3.38	4.65	3.85
SynChem	1546	28.27	19.66	8.55	6.34	1.02
InFarmatik	1373	0	34.38	0	15.29	0
Biotrend	1261	35.21	60.75	18.54	41	9.86
Prestwick	1181	8.55	59.44	3.56	34.38	1.48
Adesis	1173	28.99	8.78	17.48	3.5	0
Chemivate	1108	98.92	42.69	86.26	22.56	49.2
Endeavour	813	20.79	12.05	2.04	2.83	0
Endotherm	677	72.23	38.4	23.85	18.46	8.8
KaironKem	647	38.64	84.23	0	57.81	0
GreenPharma	647	1.24	22.41	4.83	8.19	0
AFChemPharm	617	74.07	45.38	41.79	26.74	23.03
Chess	563	1.07	22.2	0	8.53	0
Synphabase	490	1.02	41.02	0	27.55	0
Pyxis	317	97.16	12.62	37.5	7.57	0
Sinof	263	5.32	12.17	0	5.7	0
EnzoLifeSciences	202	47.03	16.83	5.88	10.4	0
Azasynth	67	100	77.61	76.92	47.76	3.13
